# Effective Oriental Magic for Analgesia: Acupuncture

**DOI:** 10.1155/2022/1451342

**Published:** 2022-03-12

**Authors:** Menglong Zhang, Lei Shi, Shizhe Deng, Bomo Sang, Junjie Chen, Bifang Zhuo, Chenyang Qin, Yuanhao Lyu, Chaoda Liu, Jianli Zhang, Zhihong Meng

**Affiliations:** First Teaching Hospital of Tianjin University of Traditional Chinese Medicine, National Clinical Research Center for Chinese Medicine Acupuncture and Moxibustion, Tianjin 300381, China

## Abstract

Pain is a kind of complex physiological and psychological symptom, which makes the person debilitated and uncomfortable. Some persistent pain is unbearable for the patients, reducing the quality of life and bringing considerable pressure to the individuals and society. Pain killers seem to be effective in analgesia for patients, but their safety and addiction are crucial issues. From the theory of traditional Chinese medicine (TCM), the blocked meridian is the main cause of pain, and effective acupuncture can play a positive analgesic effect. Acupuncture that can date back thousands of years is one of the ancient medical practices in China. Its safety and effectiveness are respected. Based on its superior safety and inferior side effects, it has been gradually recognized as a therapeutic intervention method for complementary medicine, which is also generally used to treat multiple pain diseases. It is shown by modern medical studies that neurotransmitters are the material basis for the acupuncture effect, and the effect of acupuncture analgesia is related to changes in neurotransmitters. However, the specific mechanism has not been elucidated. This review aims to comprehensively discuss the historical evolution of acupuncture analgesia, clinical research of acupuncture analgesia, comparison of acupuncture and drug therapy, the neurotransmitter mechanism of acupuncture analgesia, the effect of acupuncture manipulation on analgesia, and bibliometric analysis of acupuncture treatment for pain, to explore the superiority and related mechanism of acupuncture analgesia from different aspects, and to provide a more effective treatment for alleviating patients' pain.

## 1. Introduction

Pain is often the main compelling reason for seeking medical attention, and it can seriously affect the quality of life [1, 2]. Pain is a complex process propagated by many systems [3]. After the nociceptive stimulus is transformed into a nerve impulse, the pain sensation and pain response are produced by integrating and processing all aspects of the central nervous system (CNS) [4]. There are numerous methods to relieve pain. So far, the most effective drugs to relieve pain are opioids, and the most widely used drugs are nonsteroidal anti-inflammatory drugs (NSAIDs); at the same time, addiction, drowsiness, and other side effects could not be ignored [5, 6]. Abuse of painkillers may cause serious damage to brain function [7]. The severe opioid crisis is a tough issue that deserves more attention. It is urgent to search for some safe therapeutic approach to deal with the present emergency [8]. Due to the lack of analgesic drugs with positive curative effects but no apparent side effects, the research of alternative treatment methods has further promoted [9].

Acupuncture is also the mainstay of pain management [10]. As a therapeutic intervention approach for alternative medicine, acupuncture has gained popularity around the world. It is commonly known that acupuncture is the effective pain management from China. Today, many types of research have confirmed that acupuncture has a positive analgesic effect in treating pain [11]. A significant quantity of clinical trials has shown that the neuromodulation of acupuncture can control pain and inflammation in many diseases [12]. It has an excellent curative effect in relieving headache, neuropathic pain, lumbago, knee osteoarthritis (KOA), and other pain diseases [2]. Some international clinical guidelines suggest that the effect of acupuncture on pain is equivalent to traditional pharmacology or interventional technology [13]. Due to its positive effect and superior safety, it is more acceptable to the general population [14].

In 1997, the National Institutes of Health (NIH) recognized that acupuncture alleviates pain. Although basic research has attempted to explain the underlying mechanisms of its effects, these mechanisms have not yet been determined [15]. Acupuncture mainly exerts an analgesic effect by activating acupoints with special anatomical locations [16]. Through the stimulation of acupoints, it can produce analgesia mainly by activating neurotransmitters [17]. Although multiple types of research have attempted to elucidate its analgesic mechanism, the mechanism is still unclear. From a clinical perspective, some rigorous large-scale, multicenter randomized controlled trials ought to be implemented to investigate the mechanism of acupuncture analgesia. This review intends to introduce the history of acupuncture analgesia, the clinical research status of acupuncture analgesia, comparison of acupuncture and drug therapy, its neurotransmitter mechanism, and limitation.

## 2. Historical Evolution of Acupuncture Analgesia

In ancient times, the Chinese discovered that using stones (those sharp stones are the prototype of the current needle) to press on the painful parts of the body can relieve the pain. Along with the science and technology development, needle material has changed from stone and bone to gold, silver, copper, and iron. At present, the most popular one is stainless steel [18]. Acupuncture did not have a theoretical basis until the appearance of Huangdi Neijing (The Yellow Emperors Internal Classic) more than two thousand years ago, and the acupuncture theory is mainly recorded in its Su Wen chapter [19]. Acupuncture treatment of pain disorders has also been documented since the Huangdi Neijing period [20]. In the Ming Dynasty, the development of acupuncture reached its climax. Many doctors have offered different opinions on acupuncture and published plenty of masterpieces, but the well-known work was Compendium of Acupuncture and Moxibustion by Dr. Yang Jizhou. In his book, he recorded the manipulation and indications of acupuncture and elaborated on acupuncture analgesia in detail.

Acupuncture is an effective method of therapeutic intervention for alternative medicine that has been widely accepted around the world. Acupuncture was introduced to the West as early as the 16th century, and it was not until the 19th century that it began to be used in clinical practice by Western medicine. Acupuncture analgesia was once introduced in the condition of the use of acupuncture in surgical anesthesia. The first operation of acupuncture analgesia was reported in 1958 in China [21], and it was the result of integrated TCM and Western medicine. Until 1971, Dimond witnessed the application of acupuncture analgesia during surgery and made the first report about acupuncture analgesia in JAMA [22]. In 1973, the Lancet published an article about acupuncture analgesia. Until 1997, NIH publicly recognized acupuncture's efficacy and potential therapeutic effects in alleviating pain and multiple other disorders.

From the perspective of TCM, acupuncture is based on the theory of meridians and uses the needle to penetrate specific acupoints of the human body to cure diseases. The potential rationality of acupuncture is that diseases related to Qi (energy as considered in TCM) can be treated by stimulating related acupoints [23]. In the view of TCM, Qi is essential energy flowing through meridians and can participate in various homeostatic regulations in the human body [24]. Moreover, the analgesic effect can be achieved by stimulating Qi inside the body [25]. From the perspective of modern medicine, the underlying physiological mechanisms of acupuncture analgesia can be roughly separated into peripheral and central mechanisms, and the most complete system involved in pain management is the endogenous opioid system [17]. Changes in neurotransmitters may also be relevant to analgesia [26]. Segmental inhibition of the spinal cord produced by acupuncture may elevate the pressure pain threshold [27]. Within the modern medical system, the acupuncture theoretical system has been well-established, and scientists have made adequate progress in acupuncture for the treatment of pain disorders.

## 3. Clinical Research Condition of Acupuncture Analgesia

Since its origin in China, acupuncture has been used in more than 180 countries and regions. Forty-three diseases used for acupuncture treatment have been acknowledged by the World Health Organization (WHO) [17]. The indications of acupuncture for curing pain diseases are becoming more extensive, and the spectrum of diseases is constantly completed. Since the development of evidence-based medicine in the discipline of acupuncture, many acupuncture clinical studies have been implemented to confirm the availability of acupuncture analgesia [28, 29].

The option of the proper control group is beneficial for improving the feasibility of acupuncture research [30]. However, acupuncture is a complex intervention. It is rough to interpret the acupuncture efficacy in a single control group. The choice of the control group remains a methodological challenge in the design of acupuncture RCTs [31]. Therefore, the ideal control group is more conducive to the progress of acupuncture research. In clinical research on acupuncture treatment of pain, three control groups are usually selected. The groups include (i) needling fake acupoints (offset the correct acupuncture point); (ii) true acupoints, but using a thin needle or a very shallow depth of acupuncture (minimize the acupuncture stimulation); and (iii) placebo needle that does not penetrate the skin. Many clinical pain studies on acupuncture have shown that the analgesic effect of the acupuncture group is superior to the standard control group [32]. In acupuncture randomized studies, the curative effect of the acupuncture group and the minimal acupuncture group is better than the waiting list in the alleviation of osteoarthritic pain [33]. The placebo effects always occur at the same time as the acupuncture effects. High-quality clinical evidence indicates almost no apparent difference between true and sham acupuncture in the management of chronic low back pain [34]. In partial clinical trials of acupuncture, sham acupuncture is as effective as real acupuncture [32]. A large-scale RCT divided 857 individuals equally into the optimal acupuncture, the shallow acupuncture, and the sham acupuncture groups to treat cervical spondylosis-related neck pain. It was found that the optimal acupuncture group had the best effect, followed by the shallow acupuncture group [35]. The analgesic effects of the acupuncture and the placebo groups are superior to those of the no acupuncture groups, but placebo analgesia has only a tiny to moderate effect [36]. Some research has shown that acupuncture is available for numerous chronic pain diseases such as nonspecific musculoskeletal pain, osteoarthritis, and omalgia [37]. The effectiveness of acupuncture was better than the sham and no acupuncture group, alleviating the pain cannot be elucidated by the placebo effect barely [37]. It can also be found that acupuncture in different acupoints has a positive analgesic efficacy on sciatica treatment, and the effectiveness is better than the sham acupuncture group [38]. In alleviation of knee osteoarthritis (KOA), acupuncture has a noticeable higher cure rate than sham acupuncture, it is worth mentioning that over time, the utility of EA seems to be higher than other control groups [39]. This may be related to the frequency of acupuncture and the intensity of stimulation. The research on EA has declared that low-frequency electricity is more valid for nociceptive pain; instead, high-frequency electricity has a potent therapeutic effect for neuropathic pain [40]. Nevertheless, the frequency of acupuncture seems challenging to quantify. Although the analgesic effect of the acupuncture group was superior to the control groups, it cannot be neglected that on the management of some pain (pain in labor, migraine, and neuropathic pain), low-quality evidence suggests that acupuncture does not differ much from usual care [41–43]. Since a large number of the current RCTs provide conflicting and inconclusive evidence, the methodological shortcomings of RCTs are recurrent [43, 44].

In terms of reducing the pain effectively, acupuncture combined with other therapies also has a powerful effect. Compared with a single therapy, acupuncture combined with medical training therapy is more effective than routine care [45]. Based on traditional Western medicine, EA can relieve severe cancer pain and reduce the dose of opioid analgesics [46]. Traditional acupuncture combined with drugs is more effective than drugs alone in treating migraine [47]. Studies have shown that different acupuncture methods (actually stimulating acupoints but with different intensities) also have an analgesic effect. Twist ankle acupuncture (WAA) and auricular acupuncture (AA) have curative effects on cancer pain, and the combination of different acupuncture methods has a better analgesic effect [48]. Patients with chronic low back pain experienced a notable decrease in VAS scores after treatment with thread embedding acupuncture (TEA) combined with acupuncture, indicating that acupuncture combined with TEA has better analgesic efficacy than acupuncture alone [49].

In summary, acupuncture analgesia is widely believed to be superior to placebo needles, but a large amount of low-quality evidence has been provided clinically due to the shortcomings in RCT methodology and deficiency of rigorous trial design. However, in almost all clinical trials, the acupuncture group was superior to the usual care group, which provides abundant evidence that the effect of acupuncture analgesia is credible. Since the placebo effect is ubiquitously present in RCTs and seems to have a great connection with patient expectations, which seems inevitable and can only minimize the placebo effect, therefore further studies are needed to confirm that acupuncture is not a powerful placebo. Due to the wide range of indications, there are lots of clinical studies on MA and EA. Research on other acupuncture therapies is still insufficient. However, based on the current indications for these acupuncture treatments, to further enhance the analgesic efficacy, acupuncture combined with these therapies should also be advocated. Crucially, rigorous clinical trials must be carried out so that effective interventions for acupuncture analgesia can reasonably be provided.

## 4. Acupuncture vs Analgesic Drugs

Compared with addictive opioids, as a valid agent for pain management, nonsteroidal anti-inflammatory drugs are one of the most widely prescribed drugs [6]. However, attention should be paid to gastrointestinal complications and cardiovascular diseases associated with long-term use [50, 51]. There is no substitute for NSAIDS in some diseases, such as rheumatoid arthritis, but the major cardiovascular events they entail are even harder to avoid [52]. To minimize the harm caused by its side effects, several strategies have been adopted. Therefore, there is a need for a treatment that allows for effective pain management with fewer side effects for long-term use. Acupuncture can provide analgesia by releasing endogenous opioids with a few side effects and high safety and could be an effective alternative therapy for analgesia to the clinical use rate of drugs to avoid potential adverse events caused by medicines [53, 54].

The analgesic effect of acupuncture in certain pain disorders is as effective as NSAIDs, even has a faster and longer-lasting action in acute pain analgesia. Cho et al. divided 45 patients suffering from chronic neck pain into three groups, and after three weeks of treatment, there was no difference in VAS scores between the acupuncture-treated group and the NSAIDs-treated group, and acupuncture could provide excellent analgesia [55]. Murugesan et al. divided 157 patients with symptomatic irreversible pulpitis into three groups according to the mode of emergency pain management, and the acupuncture combined with placebo tablet group had longer-lasting analgesia and faster efficacy initiation than the sham acupuncture combined with ibuprofen group [56]. Not only acupuncture but also other acupuncture methods derived from traditional acupuncture can reduce drug dependence. Zhang et al. conducted a randomized, sham-controlled prospective study and found that auricular acupressure was analgesic and did not cause any adverse events in the treatment of KOA, and was effective in reducing the use of NSAIDs. A Systematic Review and Meta-Analysis conducted by Wu et al. found that patients treated with transcutaneous electric acupoint stimulation (TEAS) were less dependent on opioid analgesics after surgery compared to controls [57]. A large retrospective cohort study by Timothy et al. found that acupuncture shows significant advantages in complete opioid discontinuation compared with NSAIDs. Acupuncture can be used as a complementary therapy in combination with NSAIDs for better analgesia. In their study, Dingemann et al. divided 46 patients with postoperative swallowing pain into three groups, each receiving NSAIDs, and showed that the analgesic effect of the acupuncture group was better than that of the drug treatment group [58]. Interestingly, acupuncture may be the best option for pain relief when a patient cannot take NSAIDs or other analgesic medications, exerting analgesic effects even faster than medication. In treating patients with NSAIDs-tolerant dysmenorrhea, Iorno et al. showed a significant reduction in the duration of dysmenorrhea and the use of NSAIDs by acupuncture intervention, with a 74% reduction in NSAIDs [59]. Kaynar et al. found that the analgesic effect of acupuncture in urolithiasis-driven renal colic pain relief was superior to that of diclofenac and acetaminophen after ten minutes, and there was no significant difference in the analgesic effect of the three types of therapy after 120 minutes [60].

**Figure 1:**
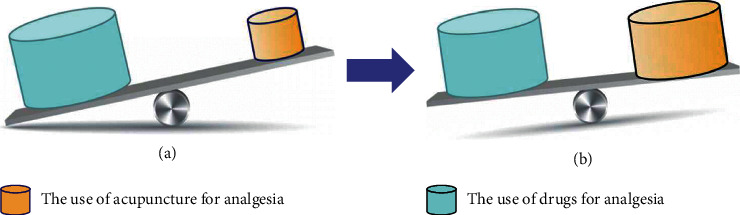
Changes in the use rate of acupuncture and drugs in the treatment of pain diseases. (a) In the past, drugs were widely used as the first choice for analgesia. (b) Based on the superiority and safety of acupuncture, acupuncture is now also used extensively as an effective analgesic therapy for various diseases. It is worth mentioning that acupuncture reduces the abuse rate of analgesics and may be able to replace painkillers for patients with some pain diseases or unable to use drugs.

As a safe analgesic therapy with low side effects and low economic burden, acupuncture has a lower incidence of adverse events than drug therapy. It even shows excellent superiority in the treatment of some diseases. For patients who cannot use NSAIDs or other analgesics, acupuncture can be an effective alternative for better pain relief. However, the effectiveness of acupuncture for analgesia is not limited to traditional MA or EA. Other related acupuncture modalities based on acupuncture theory have also demonstrated great superiority. When combined with drugs to treat pain diseases, acupuncture can enhance the analgesic effect and reduce patients' dependence on drugs and even reduce the side effects of drugs. Therefore, acupuncture may play an essential role in formulating the analgesic treatment plan, effectively ensuring patients' quality of life and lowering the ratio of drug use (Figure 1).

## 5. Mechanism of Neurotransmitters in Acupuncture Analgesia

**Figure 2:**
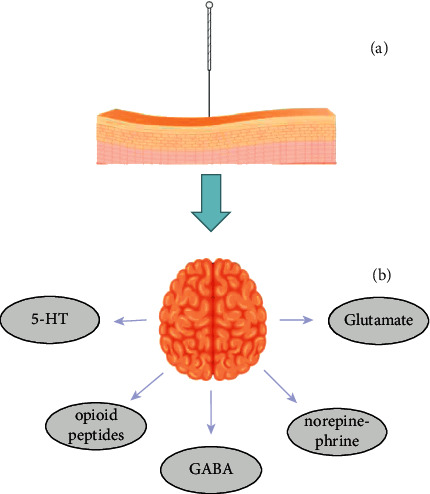
Release of neurotransmitters after acupuncture. (a) The corresponding stimulation is produced after acupuncture into the acupoint. (b) The signals induced by acupuncture are transmitted to the relevant regions of the brain, so as to release the above five neurotransmitters, and achieve the effect of analgesia through the interaction between different neurotransmitters.

Acupuncture analgesia is a comprehensive effect of transmitting the signals generated by acupuncture induction to relevant regions of the spinal cord and brain, thereby increasing and decreasing neurotransmitters to achieve the purpose of analgesia [16]. Some neurotransmitters (opioid peptides, *γ*-aminobutyric acid, norepinephrine, and 5-hydroxytryptamine) have been found to exert analgesic effects by modulating the prescribing pain modulatory pathway (Figure 2) [53]. Glutamate likewise plays a considerable role in pain modulation (Figure 2) [61].

### 5.1. Opioid Peptides

Numerous animal and clinical trials have demonstrated that acupuncture is an excellent means of analgesia. EA stimulation can release endogenous opioid peptides for positive pain management [5, 17]. Their receptors are similarly involved in pain mechanism modulation [62]. The endogenous opioid mechanism is the most well-recognized neuronal mechanism of acupuncture analgesia. There are mainly four opioid peptides: enkephalins, endomorphins, dynorphins, and nociceptin, and their *δ*, *μ*, and *κ*-opioid receptors and nociceptin peptide receptor [16]. Elucidating the endogenous response to pain is essential to optimize therapeutic action and minimize side effects [63]. EA at various frequencies may provoke different endogenous opioid mechanisms, and the healing effect of low-frequency EA is better than that of high-frequency EA [64]. In the collagenase-induced osteoarthritis (CIOA) rat model, 2 Hz EA has a better analgesic effect than 100 Hz EA [65]. The activation of the opioid peptide mechanism may be related to frequency. EA may have a superior effect by virtue of its controllable frequency advantage. Low-frequency EA can promote *β*-endorphin expression to relieve neuropathic pain [66]. At 2 Hz frequency, EA induces the release of *β*-endorphin, endomorphin, and met-enkephalin combined with *µ*- and *δ*-opioid receptors to achieve pain management. At 100 Hz frequency, dynorphin can be released and activation of *κ*-opioid receptors [67]. At different frequencies, EA may activate opioid receptors in different parts of the brain. At 2 Hz frequency, the *µ*-opioid receptors binding potential of the anterior cingulate cortex was significantly increased [68]. In the goat experiment, compared with other frequencies, EA at 60 Hz increased the pain threshold most [69]. All of this indicates that the effect of electroacupuncture is closely relevant to frequency.

In the EA treatment of inflammatory pain, activation of the peripheral cannabinoid CB2R may increase *β*-endorphin levels in inflamed tissues and combine to activate *µ*-opioid receptors to achieve analgesia [70]. Through activating *µ*-opioid receptors, the expression level of netrin-1 can be reduced to alleviate neuropathic pain caused by RTX [71]. Animal experiments on labor pain management have shown that EA can enhance the protein activation of *κ*-opioid receptors, but mainly in the lumbar spine [72]. This suggests that acupuncture analgesia may be related to the choice of acupoints. Different receptors in specific regions of the CNS may mediate different frequencies of EA.

The study of endogenous opioid peptide mechanisms is beneficial for elucidating acupuncture placebo analgesic utility. Placebo analgesia was also associated with endogenous opioid peptides [73]. The analgesic effect of the placebo can be blocked by opioid antagonists such as naloxone [74]. Studies are suggesting that the placebo effect may be related to patient expectations [75]. The placebo effect triggered by expectation can activate opioid neurotransmission and thus play an analgesic role [76]. Opioid antagonists can block pain modulation elicited by patient expectations [77].

### 5.2. *γ*-Aminobutyric Acid

*γ*-Aminobutyric acid (GABA) mainly plays an inhibitory role in the CNS. EA analgesic mechanism is closely related to GABA expression. It is well known that GABAA and GABAB can participate in pain regulation. GABAA and GABAB are the main subtypes involved in EA analgesia [78]. In the animal experiment of KOA mice, the signal pathway mediated by GABA can be involved in EA to improve pain [79]. PAG is one of the main centers of descending pain suppression system [80]. GABA released in the PAG may involve pain management. Under EA treatment, the glutamate declined in the hippocampus, and the GABA enhanced in the PAG, due to the increase of GABA receptors, 15 Hz EA but not 2 Hz or 50 Hz can relieve mechanical and thermal hyperalgesia pain [81]. In the experiment of EA treatment of neuralgia in rats, under 2 Hz and 15HZ EA, the level of GABAA receptor in the spinal cord of rats was higher than that of the sham acupuncture group [82]. After EA treatment, the GABA concentration in DRG increased, and EA can alleviate incisional neck pain by upregulating GABA expression in DRGs [83]. EA seems to upregulate the GABA expression and its receptors in spinal cord DHs to have a good analgesic effect on rats with incision neck pain [84]. GABA may be involved in compensatory enhanced acupuncture analgesia. After EA treatment, increased GABA exerts an analgesic effect in FM patients [85]. GABAA receptor *γ*-2 subunit participates in EA alleviating neuropathic pain [86]. Other types of research have proved that EA can induce the release of endogenous endorphins and inhibit the release of GABA by activating *µ*-opioid receptors of GABAergic neurons [87]. Consequently, GABA participates in the pain management of EA, and the frequency and area of EA may also affect the production of GABA.

### 5.3. Norepinephrine

Studies have shown that norepinephrine combined with *α*2-adrenoceptor can achieve analgesic effects [88]. EA can downregulate pain inhibitory pathways by enhancing the release of norepinephrine [89]. The involvement of norepinephrine in pain relief is mediated by the stimulation of adrenergic receptors on inflammatory cells that release *β*-endophilin to achieve analgesia [90]. The analgesic effect of noradrenergic in the dorsal horn of the spinal cord is probably through activation of the inhibitory factor *α*2-adrenergic receptor [91]. EA controls the transmission of pain messages by activating projection to spinal noradrenergic neurons [92]. Tolerance is similarly observed in EA analgesia. Cross-tolerance may develop between norepinephrine and EA, probably owing to the large secretion of norepinephrine in the brain, acting through *α*-receptors, against EA analgesia [93]. Its tolerance should be thoroughly studied to maximize the analgesic effect. Further research is needed on the mechanism of action of norepinephrine.

### 5.4. 5-Hydroxytryptamine

5-Hydroxytryptamine (5-HT) is generally renowned as serotonin. It is the neurotransmitter of the descending inhibitory system of the brainstem involved in analgesia. 5-HT secretion increased in the brain during acupuncture analgesia [94]. It is mainly produced in the rostroventromedial medulla (RVM) to the spinal cord and exerts bidirectional modulatory effects in descending facilitatory and inhibitory pathways [53]. 5-HT is a pain mediator in the periphery, which can decrease the secretion of pain-related factor 5-HT after acupuncture [95]. 5-HT of descending pain regulatory system participates in acupuncture analgesia. Different types of research have shown that surgical pain can be alleviated by upregulation of 5-HT receptors (5-HT1AR and 5-HT2AR) by EA [96]. In a rat model of recurrent migraine, 5-HT levels in the plasma of the EA-treated group were higher in the RVM and trigeminal nucleus caudalis regions than those of the other treated groups [97]. After EA treatment, the number of neurons and the relative protein expression of 5-HT7R in migraine rats were significantly decreased [98]. Related rats' pain experiments showed that 5-HT1AR and 5-HT3AR could participate in mediating EA analgesia [99]. Osteoarthritis-induced pain can be suppressed by EA enhancement of spinal 5-HT2A/2C receptor activity [100]. In the model of CIOA rats, 5-HT1R and 5-HT3R can reduce the analgesic effect of 2 Hz EA [65]. EA at 100 Hz reduced pain and upregulated 5-HT expression in DRN of reserpine injected rats [101]. Different matching points may have different efficacies. Liu et al. [102] measured the concentration of 5-HT and 5-HT4R in chronic visceral hypersensitivity rats after EA stimulation by ELISA, and the results suggested that EA could improve the pain threshold, decrease the concentration of 5-HT, and increase the concentration of 5-HT4R. However, it makes no difference in the concentration of 5-HT3R. These studies clearly show that serotonin is involved in acupuncture analgesia, but the corresponding matching points should be selected for better analgesic effects. Moreover, EA analgesia at different frequencies may be antagonized or enhanced by 5-HT. The choice of the corresponding frequency is also particularly significant.

### 5.5. Glutamate

The most widely spread neurotransmitter in the CNS is glutamate, which plays a vital role in excitatory ascending pathways. It is a crucial excitatory neurotransmitter that efferents from the dorsal horn of the spinal cord [103]. Glutamate and its receptors N-methyl-D-aspartate receptor (NMDAR) participate in the transmission and integration of pain messages at the spinal level [16]. Glutamate can induce CNS sensitization by activating its receptors to act as an analgesic. Acupuncture can achieve an analgesic effect by downregulating glutamate in the ascending excitation pathway [53]. Glutamatergic pathways may induce acupuncture analgesia [104]. Central sensitized NMDAR may participate in spinal cord pain [105]. Studies have suggested that EA combined with NMDA antagonists produces a stronger antihyperalgesic effect [106]. In the CCI-induced neuropathic pain model, glutamate is reduced in the hippocampal region of PAG rats under EA analgesic treatment [81]. Although the acupuncture method is different, it seems to play the same analgesic effect. In a rat model of neuropathic pain, WAA reduced pain sensitivity possibly by inhibiting the expression of Glu and P-NMDAR1 in the spinal dorsal horn [107]. The modulation of glutamate and its receptor content by acupuncture is beneficial for optimizing the analgesic effect of acupuncture.

In conclusion, the frequency of EA is fixed and controllable. The release of neurotransmitters seems to be related to a fixed frequency, with different frequencies releasing different neurotransmitters. The analgesic effect is closely related to the quantity of acupuncture stimulation. Most of the current studies on acupuncture analgesia are based on animal models, and the choice of acupuncture points in different models is diverse. Whether the different anatomical locations affect the release of different neurotransmitters remains to be clarified. The neurotransmitters released are different when acupuncture is applied to rapid analgesia and slow analgesia. How to shift the analgesic phase and release neurotransmitters to maximize the analgesic effect remains to be further investigated. There are other mechanisms of acupuncture analgesia. How to combine multiple mechanisms to achieve the best analgesic effect deserves our attention.

## 6. Effect of Acupuncture Manipulation on Analgesia

Plenty of clinical trials and molecular mechanism studies have proved that acupuncture analgesia is indeed effective. But the analgesic effect of acupuncture still has limitations, for instance, placebo as mentioned above effect and the inadequacy of RCTs design such as the design of an effective control group. The specificity and quantity of acupuncture also deserve further exploration.

The manipulation of acupuncture closely relates to physicians' personal clinical experience, and it is hard to achieve standardization. Heterogeneity among practitioners may bias the analgesic effect of acupuncture. There are high-quality meta-analyses of acupuncture trials for chronic pain that demonstrate a more significant variation in treatment efficacy among different practitioners than would be expected by chance [108]. Between the differences in the manipulation of practitioners, the efficacy achieved with acupuncture may also be biased. Acupuncture techniques have various characteristics and have commonalities, but their effectiveness is different [109]. Li et al. [110] found that different acupuncture manipulations may have different effects on blood perfusion. Using five different acupuncture methods to treat patients with KOA found that EA and fire acupuncture were more effective than other methods [111]. The right acupoint is significant to acupuncture. In treating patients with ischemic stroke, changes in brain activity by fMRI contrast between acupuncture Wai Guan (SJ5) and sham acupoints indicated a remarkable decrease in the BOLD signal of the right BA5 after acupuncture SJ5 compared with sham acupoints [112]. In the formalin-induced pain rat model, acupunctures ST36, SP9, and BL60 had a better analgesic effect than the control group, and BL60 had the most significant effect [113]. Studies have shown that acupoints have specificity in treating migraine, and the effect of acupuncture at SJ5, GB34, and GB20 is better than that of the control group [114]. Bias due to the specificity of acupuncture, such as the choice of practitioners, acupuncture methods, and acupoints, should be minimized, and a large number of studies are necessary to confirm the specificity of acupuncture.

The quantity of acupuncture is related to the number of acupoints, the number of needles, the frequency of acupuncture, and the duration of treatment [11, 115]. High-quality RCTs suggest that an eight-week thrice-weekly EA may have the optimum effectiveness in reducing pain in patients with KOA [39]. Meta-analyses suggest that acupuncture is a positive medical method for limb pain, but it takes five or eleven weeks to achieve maximum effect [11]. The curative effect of acupuncture can be achieved by rotating the needle. After acupuncture at SJ5, it was shown by fMRI that the BOLD signal was higher when the needle was rotated than when it was not rotated [112]. The underlying mechanism of the persistent effect of acupuncture remains unclear.

## 7. Bibliometric Analysis of Acupuncture in the Treatment of Pain

There are a few bibliometric analyses on acupuncture analgesia. In recent years, studies related to acupuncture analgesia have gradually increased. We attempted to analyze the progress and research trend of acupuncture analgesia by bibliometric method to observe the discipline's current research hotspots and frontier areas. All data were retrieved from the Web of Science on December 31, 2021, with the search terms (acupuncture) AND (pain). We selected publications from the last decade, with publication dates from January 1, 2011, to December 31, 2021. A total of 4781 articles were included in the bibliometric analysis after excluding articles that could not be used as full-text duplicate publications. We used VOSviewer v.1.6.17 to carry out descriptive statistical analysis on publications from journals, keywords, authors, and countries. We used Excel 2019 to analyze the trend of literature published in the past decade.

### 7.1. Analysis of Annual Publications

**Figure 3:**
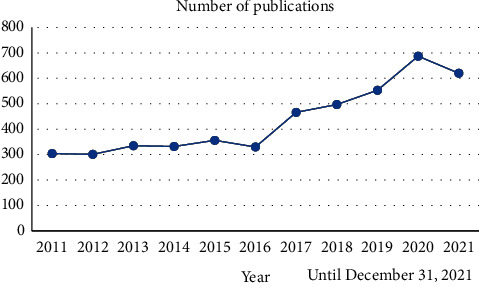
Changes in the number of acupuncture for pain diseases' literature publications from 2011 to 2021 until December 31, 2021.

A total of 4781 papers were identified for this study. The number of articles published each year is shown in Figure 3. Although the number of publications fluctuated, it generally showed an increasing trend yearly, from only 304 in 2011 to 687 in 2020. Compared with 2020, the number of publications in 2021 reduced to 620.

### 7.2. Analysis of Journals

**Figure 4:**
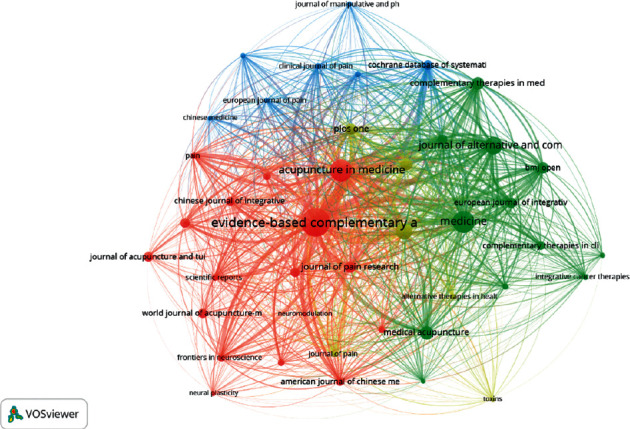
Bibliometric coupling of journals.

4781 articles on acupuncture treatment for pain came from 1043 journals. Among them, Evidence-Based Complementary and Alternative Medicine published the largest number of publications (7.8%), followed by Medicine (4.8%) and Acupuncture in Medicine (4.8%; Table 1). Table 1 lists the impact factor (IF) and publishing countries of the top ten journals. Most of the journals are published in England and the United States. The top ten journals with the highest IF are BMC Complementary and Alternative Medicine, with an IF of 3.659.  Figure 4 shows the bibliometric coupling of journals. This indicated the degree of association between different journals.

### 7.3. Analysis of Keywords

**Figure 5:**
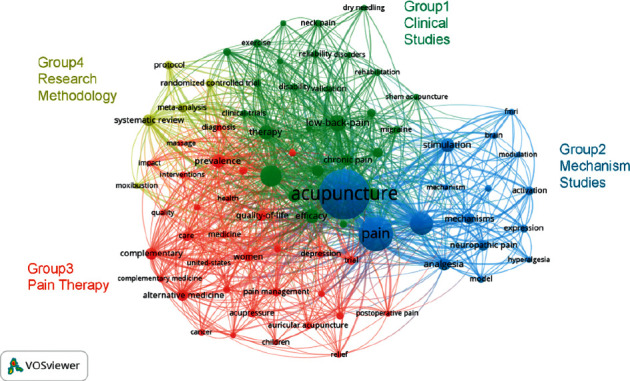
Network map of keywords of acupuncture and pain (divided into four groups).

Based on the frequency of keywords, the current research area can be identified. The keywords in 4781 articles were analyzed by VOSviewer, and a total of 11,483 keywords were mentioned in all articles of which 80 were mentioned more than 85 times. We divided these keywords into four groups by VOSviewer. As shown in Figure 5, different colors represent different groups and research directions. Group 1 represented clinical studies, in which a total of 27 keywords were mentioned, of which the most frequent keyword was management (689 times), followed by low back pain (433 times). Group 2 represented mechanism studies, in which a total of 16 keywords were mentioned, of which the most frequent keyword was acupuncture (2674 times), followed by pain (1420 times). Group 3 represented pain therapy, in which a total of 32 keywords were mentioned, of which the most frequent keyword was prevalence (339 times), followed by complementary (253 times). Group 4 represented research methodology, in which a total of 5 keywords were mentioned, of which the most frequent keyword was systematic review (262 times), followed by meta-analysis (166 times).

### 7.4. Analysis of Authors

**Figure 6:**
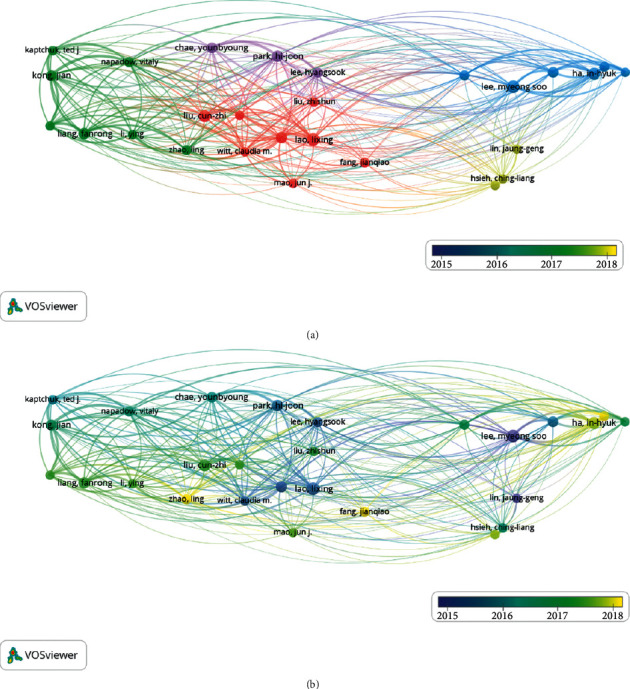
Network map of authors. (a) Authors with more than 25 articles. (b) Year of author's publication in the journal.

We found a total of 18,841 authors by VOSviewer, of whom 27 authors have more than 25 articles. From Figure 6, we found that the top five authors in terms of the number of articles published were Lao Lixing (46 articles), Lee Myeong soo (45 articles), Liu Cunzhi (42 articles), Ha In-hyuk (41 articles), Park Hi-joon (40 articles), and Macpherson Hugh (40 articles). We used VOSviewer to analyze the author's publication year, and the lighter the color is, the closer the publication year is to the present.

### 7.5. Analysis of Countries

**Figure 7:**
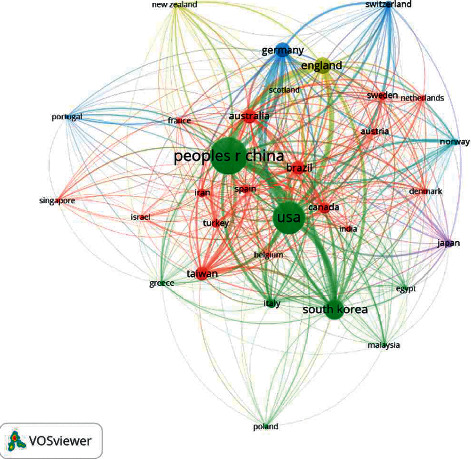
Network map of countries with at least 20 publications.

A total of 91 countries have published publications on acupuncture treatment for the pain of which 32 countries have published more than 20 articles. As shown in Figure 7, the top five countries in terms of the number of publications were China (1577 articles), the United States (1205 articles), South Korea (465 articles), England (330 articles), and Germany (258 articles). As the birthplace of acupuncture, China had the largest number of publications, although the bibliometric analysis in this paper did not include Chinese journals.

## 8. Discussion

Acupuncture is an effective method of analgesia. Since acupuncture analgesia was discovered, its effectiveness has been confirmed by numerous clinical and molecular mechanism studies. Acupuncture has been widely applied to alleviate several pain diseases and incorporated into guidelines, and it is an effective method of coping with the opioid crisis and is widely respected due to its safety, low price, and low addiction. The use of acupuncture helps to increase the levels of neurotransmitters in the body, which can be targeted for the treatment of different pain disorders according to different activation mechanisms while maximizing the analgesic effect according to their quantity requirements. However, acupuncture analgesia still suffers from the following deficiencies: (1) Acupuncture can be used to treat various pain diseases, but the spectrum of diseases has not been well defined, and its indications should be further expanded. (2) The design of RCTs related to acupuncture is still flawed, and how to improve the design methods of RCTs to avoid bias is also an urgent issue to be solved. The placebo effect seems to affect the essence of acupuncture analgesia, and avoiding the placebo effect requires further examination. (3) Whether acupuncture can be an effective alternative therapy to drugs in the treatment of certain diseases or to develop individualized treatment plans based on acupuncture analgesia. (4) The analgesic mechanisms of the different acupuncture modalities have not been elucidated, but most of them seem to be based on the same neurotransmitter mechanism. Different frequencies of acupuncture can lead to the activation of neurotransmitters at different sites. The related mechanism has not been clarified. The mechanism of action of different neurotransmitters for analgesia also needs to be further demonstrated. (5) The choice of different acupoints would induce different analgesic efficacy, and the variability between acupoints remains to be clarified, and the choice of the correct acupoint should be based on a large body of evidence. (6) How to avoid bias produced by acupuncture specificity remains to be addressed, and the criteria for acupuncture quantification are unclear. (7) Through the literature measurement tool, we can clarify the current research trend and the cross-research between disciplines and even countries, which is significantly beneficial to the research progress of acupuncture treatment of pain, but there are still deficiencies. The literature selected in this paper is in English, while other languages are not included, which may lead to bias in some aspects. If it includes the literature in Chinese or other languages, it may make the analysis results more comprehensive. Second, the results of the bibliometric analysis may be subjective, and the results may be different due to the different settings of software applications. However, the analysis of keywords and publications in this paper can accurately determine the current prominent researchers and research hotspots, which is conducive to the induction and research of acupuncture treatment for pain. In a word, effective analytical methods and considerable basic and clinical research are still vital to confirm the related mechanism of acupuncture analgesia, thereby providing an effective means of diagnosis and treatment for the medical service.

## Figures and Tables

**Table 1:** The 10 journals publishing the highest number of articles about acupuncture treatment for pain.

Ranking	Journal title	Records (*n*)	IF2020^a^	Country	% (of 4781)
1	Evidence-Based Complementary and Alternative Medicine	375	2.629	United States	7.8
2	Medicine	233	1.889	United States	4.8
3	Acupuncture in Medicine	232	2.267	England	4.8
4	Journal of Alternative and Complementary Medicine	155	2.579	United States	3.2
5	Trials	135	2.279	United States	2.8
6	Complementary Therapies in Medicine	88	2.446	United States	1.8
7	BMC Complementary and Alternative Medicine	81	3.659	England	1.6
8	PLoS One	75	3.24	United States	1.5
9	Journal of Pain Research	71	3.133	England	1.4
10	BMJ open	68	2.692	England	1.4

Notes: ^a^IF in Table 1 according to Journal Citation Reports (2020).
